# Identification and Characterization of a Serious Multidrug Resistant *Stenotrophomonas maltophilia* Strain in China

**DOI:** 10.1155/2015/580240

**Published:** 2015-01-14

**Authors:** Yan Zhao, Wenkai Niu, Yanxia Sun, Huaijie Hao, Dong Yu, Guangyang Xu, Xueyi Shang, Xueping Tang, Sijing Lu, Junjie Yue, Yan Li

**Affiliations:** ^1^Department of Critical Care Medicine, Affiliated Hospital of Academy of Military Medical Science, Beijing 100071, China; ^2^The Institute of Microbiology and Epidemiology, Academy of Military Medical Sciences, Beijing 100071, China; ^3^Department of Respiratory Diseases, The First Affiliated Hospital of Liaoning Medical University, Jinzhou, Liaoning 121001, China; ^4^Jinan Fourth People's Hospital, Jinan 250031, Shandong, China; ^5^Beijing Institute of Biotechnology, Beijing 100071, China

## Abstract

An *S. maltophilia* strain named WJ66 was isolated from a patient; WJ66 showed resistance to more antibiotics than the other *S. maltophilia* strains. This bacteraemia is resistant to sulphonamides, or fluoroquinolones, while the representative strain of *S. maltophilia*, K279a, is sensitive to both. To explore drug resistance determinants of this strain, the draft genome sequence of WJ66 was determined and compared to other *S. maltophilia* sequences. Genome sequencing and genome-wide evolutionary analysis revealed that WJ66 was highly homologous with the strain K279a, but strain WJ66 contained additional antibiotic resistance genes. Further analysis confirmed that strain WJ66 contained an amino acid substitution (Q83L) in fluoroquinolone target GyrA and carried a class 1 integron, with an *aadA2* gene in the resistance gene cassette. Homology analysis from the pathogen-host interaction database showed that strain WJ66 lacks raxST and raxA, which is consistent with K279a. Comparative genomic analyses revealed that subtle nucleotide differences contribute to various significant phenotypes in close genetic relationship strains.

## 1. Introduction 

*Stenotrophomonas maltophilia (S. maltophilia)* is an aerobic Gram-negative bacillus ubiquitous in the natural and hospital environment [1]. In addition,* S. maltophilia* is emerging as a global multidrug resistant (MDR) human opportunistic pathogen. In a clinical environment,* S. maltophilia* has been isolated from several sources, including the suction system tubing of dental chair units, contaminated endoscopes, and tap water, all of which present possible patient exposure sources.* S. maltophilia* contaminated central venous catheters and tap water faucets have been implicated in cutaneous and soft tissue infections in patients with neutropenia [2].

*S. maltophilia* is most commonly associated with respiratory and other serious infections, especially in immunocompromised patients [3]. In polymicrobial infections,* S. maltophilia* was present in 45.8% of patients [4].* S. maltophilia* is being isolated with increasing frequency from patients with intensive care unit (ICU) associated infections [5]. ICU-acquired infections related to* S. maltophilia* were independently associated with increased morbidity and mortality [6, 7]. Specifically, early reports showed that this important nosocomial pathogen is associated with crude mortality rates ranging from 14 to 69% in patients with bacteraemia and the ICU mortality rate attributed to bacteraemia was 16.7% according to an 11 years review [4, 8, 9].

Since the first genome of* S. maltophilia* K279a was reported in 2008 [10]; bioprojects of 48* S. maltophilia* strains can be found in NCBI. However, the majority of these strains are not available.

Here, we isolated a* S. maltophilia* strain from a human blood sample, named WJ66. This strain exhibited resistance to more antibiotics than the other* S. maltophilia* strains. Drug resistance test showed that* S. maltophilia* WJ66 is resistant to nearly all drugs, including sulphonamides and fluoroquinolones. We present the draft genome sequence of* S. maltophilia* WJ66 and conduct a comparison genomic and phenotypic analysis. Genome sequencing revealed that strain WJ66 contained additional antibiotic resistance genes and an amino acid substitution (Q83L) in fluoroquinolone target GyrA and carried a class 1 integron, with an* aadA2* gene in the resistance gene cassette. This paper may lead to a better understanding of the correlation between pathogenic traits and specific genes or gene clusters of* S. maltophilia*.

## 2. Results and Discussion

### 2.1. Bacterial Strains and Drug Resistance

Blood cultures and* in vitro* susceptibility testing confirmed the presence of a multidrug resistant* S. maltophilia* isolate, termed WJ66, in an infected patient. The patient was an elderly man in ICU with underlying diseases including type 2 diabetes, diabetic nephropathy, chronic renal insufficiency, uraemia, and a lung infection. Clinical tests showed that the isolates were resistant to nearly all drugs available for treatment, including sulphonamides and fluoroquinolones [8, 11]. Multilocus sequence typing (MLST) sequencing showed that* S. maltophilia* WJ66 belongs to ST31 (1, 4, 12, 6, 6, 22, and 7 for atpD, gapA, guaA, mutM, nuoD, ppsA, and recA).

*In Vitro* 96-well plate susceptibility results showed that* S. maltophilia* WJ66 was resistant to ampicillin-sulbactam (>64–32 *μ*g/mL), piperacillin-tazobactam (>256–4 *μ*g/mL), doxycycline (>32 *μ*g/mL), trimethoprim-sulfamethoxazole (32–608 *μ*g/mL), ciprofloxacin (>16 *μ*g/mL), levofloxacin (>16 *μ*g/mL), polymyxin (4 *μ*g/mL), and amikacin (64 *μ*g/mL) and moderately resistant to minocycline (8 *μ*g/mL) and tigecycline (16 *μ*g/mL).* S. maltophilia* K279a is sensitive to minocycline (0.25 *μ*g/mL), trimethoprim-sulfamethoxazole (1–9 *μ*g/mL), ciprofloxacin (2 *μ*g/mL), and levofloxacin (2 *μ*g/mL) and resistant to amikacin (64 *μ*g/mL), gentamicin (16 *μ*g/mL), piperacillin-tazobactam (256–4 *μ*g/mL), and tetracycline (16 *μ*g/mL) according to CLSI agar dilution MICs of antimicrobials [12].

We obtained* S. maltophilia* K279a from the ATCC and examined the drug resistant differences by Etest.  Table 1 indicates that both K279a and WJ66 are resistant to imipenem, doxycycline, and tetracycline. K279a is sensitive to polymyxin, while WJ66 is moderately sensitive to polymyxin. Levofloxacin and trimethoprim-sulfamethoxazole are two of the few drugs that are generally efficacious against clinical* S. maltophilia* infections. K279a is sensitive to both these drugs, but WJ66 is resistant. The mechanisms of this resistance will be described in the following sections. Susceptibility results of control* Escherichia coli* strains (ATCC25922) and* Pseudomonas aeruginosa* (ATCC27853) are in line with CLSI standards.

### 2.2. Total Genome Overview of* S. maltophilia *WJ66

The draft genomic sequence of* S. maltophilia* WJ66 was obtained by an Illumina Hiseq 2000. A total of 258 contigs, covering a total of 4,642,973 bp, were generated. The average length of a contig is 17,996 bp with a G + C content of 66.48%. The N50 contig size was 33,808 bp. The genome includes 4,392 predicted open reading frames (ORFs) and 3 ribosomal RNAs (rRNAs) and 61 transfer RNAs (tRNAs) (Table 2). The genome sequence of WJ66 makes it possible for a comparative and functional genomic analysis of* S. maltophilia* isolates.

### 2.3. Phylogenetic Relationships among Sequenced* S. maltophilia* Strains Using Whole Genome Comparisons

**Figure 1:**
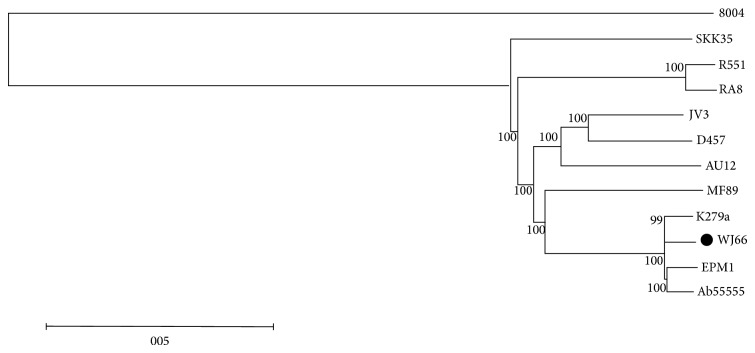
Evolutionary relationships among* S. maltophilia* strains. Neighbour-joining phylogram of* S. maltophilia*. Neighbour-joining phylogenetic tree was constructed based on a single copy gene family with the bootstrap set to 1000. Sequences from* Xanthomonas campestris* pv.* campestris* strain 8004 were used as an outgroup. The black spot indicates* S. maltophilia* WJ66. R551 and AU12 are short for R551-3 and AU12-09, respectively.

The* S. maltophilia* WJ66 genomic sequence was compared with 11 other* S. maltophilia* strains, whosegenomic sequence data are in the GenBank database.  Figure 1 shows the phylogenetic relationships between different* S. maltophilia* isolate genomes. We took the* Xanthomonas campestris* pv.* campestris* (*Xcc*) stain 8004 as an outgroup construct for the phylogenetic tree. WJ66, K279A, EPM1, and Ab 55555 are on the same branch, indicating that they are highly homologous. Strain K279a is a blood sample isolate from the United Kingdom [10], strain WJ66 is also a blood sample isolate from China, strain Ab55555 is an American isolate, and strain EPM1 was found during the sequencing of two human-derived strains of* Giardia duodenalis* [13]. The environmental isolate MF89, an oyster-associated bacteria, shows potential for crude oil hydrocarbon degradation [14] and is the most closely related to the above four isolates. In addition, JV3, D457, and AU12-09 are close relatives with each other. D457 is a clinical isolate [15] and AU12-09 is an intravascular catheters isolate [16]. Homologous strains R551-3 and RA8 were isolated from the poplar* Populus trichocarpa* [17] and from sewage plant effluent [18], respectively. SKK35 is ulcer swab isolate [9]. Overall, the* S. maltophilia* strains can be divided into two groups: the clinical isolates and the environmental isolates. The mutual affinity within groups is closer than the relationship between the groups. As the* Xcc* strain 8004 is the only non-*S. maltophilia* strain, the genomic differences between* Xcc* and* S. maltophilia* are the largest.

### 2.4. Comparing the Genomes of* S. maltophilia* WJ66 and K279a

**Figure 2:**
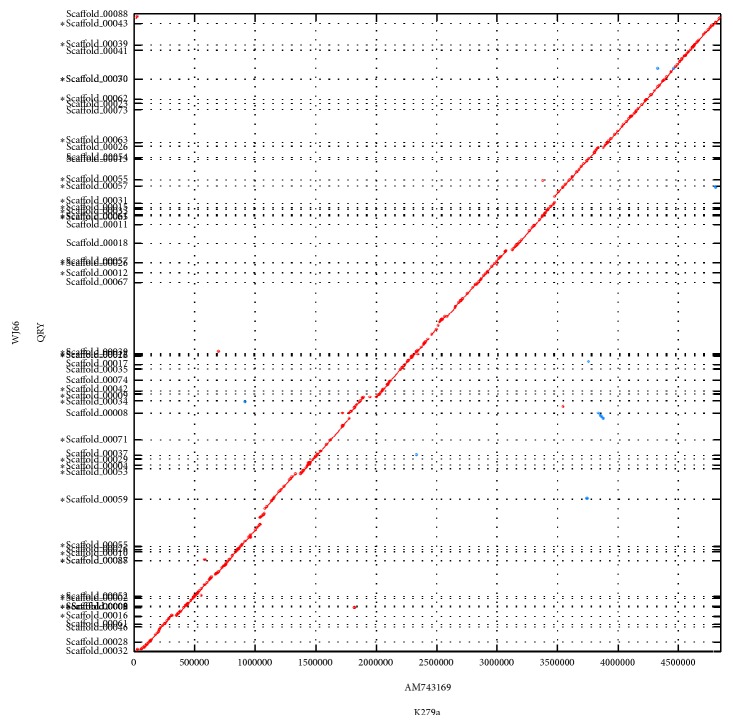
Genome alignment of* S. maltophilia* WJ66 and K279a. Line figures depict the results of whole genome alignment results using MUMmer. The query genomic sequence was the strain WJ66, and the subject genome was K279a. The red and blue lines represent direct reverse alignments, respectively.

Because* S. maltophilia* strains WJ66 and K279a exist in the same isolated background and are closely related, more comparison need to be performed. Global alignments were performed by the MUMER software [19], and the results showed a large homology between WJ66 and K279a (Figure 2).

### 2.5. The Comprehensive Antibiotic Resistance Database (CARD)

Within the CARD, 3411 genes are tagged for antibiotic molecule, biosynthesis, resistance, or inhibition of resistance, and 1859 genes were specifically annotated for antibiotic resistance. The Resistance Gene Identifier (RGI) version 2 provides an automated annotation of DNA sequences based upon the data available in the CARD, thus providing a prediction of antibiotic resistance genes [20].

**Figure 3:**
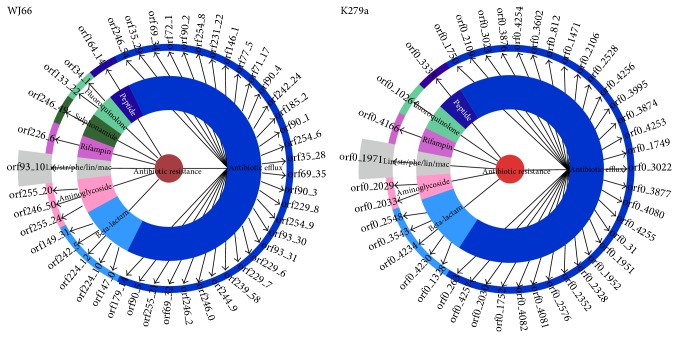
Antibiotic resistance genes in the* S. maltophilia* WJ66 and K279a genomes. Contigs were uploaded to RGI, autoannotation results are as follows: most of the resistance genes are antibiotic efflux approximately (32/31), beta-lactam resistance (5/4), aminoglycoside resistance (3/3), lin/str/phe/lin/mac (1/1), rifampin resistance (1/1), sulphonamide resistance (1/0), fluoroquinolone resistance (2/1), and peptide (1/1). The reference website is http://arpcard.mcmaster.ca/.

The autoannotation results of the WJ66 and K279a contigs are shown in Figure 3. Most of the resistance genes are antibiotic efflux genes (32 and 31 genes in WJ66 and K279a, resp.), beta-lactam resistance genes (5 and 4 genes in WJ66 and K279a, resp.), aminoglycoside resistance genes (3 and 2 genes in WJ66 and K279a, resp.), lin/str/phe/lin/mac (linezolid/streptogramin/phenicol/lincosamide/macrolide) resistance gene (1 gene in WJ66 and K279a, resp.), a rifampin resistance gene (1 gene in WJ66 and K279a, resp.), a sulphonamide resistance gene (1 gene in WJ66), fluoroquinolone resistance genes (2 genes and 1 genes in WJ66 and K279a, resp.), and a peptide (1 gene in WJ66 and K279a). This analysis reveals the resistance strength of WJ66.

Open reading frames (ORFs) obtained from the GeneMarks annotation were subjected to BLAST analysis against the CARD. This approach highlighted the presence of 255 genes related to antibiotic resistance. The largest proportion of homology to antibiotic resistance genes was found to be efflux pump genes (approximately 41 homologous genes), the beta-lactamase gene (33 homologous genes), and quinolone resistance related genes (7 homologous genes). By comparing with the CARD, we found that WJ66_04174 and WJ66_01359 codes for the* qepA* gene, whose gene product is a quinolone efflux pump, are homologous with an antibiotic resistance gene in* E. coli* 1520 genome. The protein QnrB encoded by WJ66_00023 is a* S. maltophilia* inherent quinolone resistance gene. WJ66_04112 (*gyrA*), WJ66_03278 (*gyrB*), WJ66_01551 (*parC*), WJ66_03996 (*parC*), and WJ66_03111 (*parE*) were annotated as fluoroquinolone target genes, and mutation in these genes causes resistance to quinolones. GMS_00385 encodes dihydropteroate synthetase type 1 (*sul1*); downstream* sul1* is GMS_00386 (WJ66_01310) coding an* aadA2* aminoglycoside acetyltransferase; these two genes are homologous with that found in* Salmonella enterica* subsp.* enterica* serovar Typhimurium DT104. Advanced analyses found that* sul1* and* aadA2* are members of a class 1 integron and were not predicted by GeneMark analysis. Moreover, the two genes are 100% identical with latter strain Typhimurium DT104, indicating that they were probably transferred from another bacterium by mobile elements.

### 2.6. The Trimethoprim-Sulfamethoxazole Associated Integron

In many Gram-negative bacteria, integrons carrying antibiotic resistance genes have been identified by mobile elements such as transposons and plasmids that facilitate the transfer of antibiotic resistance genes between different species. In the present study, the distribution of class 1 and 2 integrons was examined in a collection of clinical* S. maltophilia* isolates [21].

Integrons were initially described at the end of the 1980s [22]. This family of elements appears formally distinct from other known mobile DNA elements such as transposons. Class 1 integrons play an important role in the emergence and spread of antimicrobial resistance determinants. Typically, class 1 integrons have three distinct genetic regions of which two are highly conserved, the 5′-conserved segment (5′-CS) and the 3′-conserved segment (3′-CS) flank the central variable region where the gene cassettes are located. The 5′-CS includes the integrase* intI1* gene, the recombination site* attI*1, and the promoters Pc and P2 when present. The 3′-CS consists of the* qacEΔ1* gene, which encodes an incomplete version of a protein that mediates the resistance to certain detergents, the* sul1* gene, which encodes resistance to sulphonamides, and an open reading frame of unknown function orf5 [23].

Although most of class 1 integrons have this classic structure, an increasing number of class 1 integrons, related to trimethoprim-sulphamethoxazole resistance, with different structures have been reported [24, 25]. Although trimethoprim-sulfamethoxazole is considered the drug of choice for treating* S. maltophilia* infections, 25% of the isolates were resistant [21].

**Figure 4:**
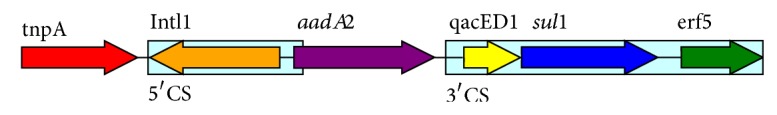
Class 1 integron in* S. maltophilia* WJ66. The* S. maltophilia* WJ66 genome carries a detectable class 1 integron;* aadA2* (resistance to aminoglycosides) was found as the only gene in the resistance gene cassette.

The WJ66 strain carries a detectable class 1 integron, whereas class 2 and class 3 integrons were not identified. In addition to the* sul1* gene encoding a sulphonamide-resistant dihydrofolate synthase, the* aadA2* gene, which encodes an aminoglycoside acetyltransferase giving resistance to aminoglycosides including amikacin, was identified as the only other gene in the resistance gene cassette (Figure 4). More details about the integron can be found in supplementary data 1 (see Supplementary Materials available online at http://dx.doi.org/10.1155/2015/580240).

There are 24 different* S. maltophilia* integrons reported in the NCBI. Therefore, we compared class 1 integron in WJ66 with other class 1 integrons of* S. maltophilia* (22/24). Gene orders in cassettes are as follows:* aadB-aac*(*6*)*-II-blaCARB-8* (GQ866976.1),* aadB-aadA1* (HQ914241.1),* aacA4-blaIMP-25-blaOXA-30-blacatB3* (GU944726.1),* blaVIM-2-aac*(*6*′)*-II* (KF471098.1),* qacL-aadB-cmlA-aadA2* (KF556708.1),* qacK-aac6-aac6* (KF556707.1),* aac6* (KF556705.1),* qacK* (KF556704.1),* aadB-aadA2* (JN108896.1),* dhfra1* (HQ584988.1),* aac*(*6*′)*-ib-cr* (HQ438047.1),* arr-3-aac* (GU137303.1),* dfrA12-aadA2* (GQ981416.1),* dfrA17-aadA5* (GQ924479.1),* aacA 4-catB8-aadA1* (GQ906532.1),* dfrA15* (JN108895.1),* aac*(*6*′)*-Ib-cr* (EF210035.1),* arr3-dfrA27* (KC748137.1),* aadB* (JX560784.1), P2 promoter-*aacA4-aadA2* (GQ896541.1),* aacA4 *(GQ502669.1),* smr* (AF406792.1), and* aadA1* (HQ832470.1).

Because class 1 integron has had a major role in the spread of antibiotic resistance and has led to worldwide difficulties in controlling bacterial infection, understanding the origin of these elements is important for the practical control of antibiotic resistance and for exploring the means by which bacterial lateral gene transfer can seriously impact, and be impacted by, human activities.

### 2.7. The Fluoroquinolone Levofloxacin Resistance Associated Gene

Quinolones are synthetic antibacterial reagents with broad-spectrum activity. They inhibit the enzyme topoisomerase II, a DNA gyrase that is necessary for the replication of the microorganism. Topoisomerase II enzyme produces a negative DNA supercoil on DNA, which permits transcription or replication. By inhibiting this enzyme, DNA replication and transcription are blocked.

The earliest quinolone, nalidixic acid, was first used in a clinical setting in 1962. The targets of quinolone action are the essential bacterial enzymes DNA gyrase, as stated above, and DNA topoisomerase IV. Both are large, complex enzymes composed of 2 pairs of subunits. The subunits of DNA gyrase are GyrA, a 97-kDa protein encoded by the* gyrA* gene, and GyrB, a 90-kDa protein encoded by the* gyrB* gene. The subunits of topoisomerase IV are ParC (75 kDa) and ParE (70 kDa).

One of the mechanisms of quinolone resistance is the accumulation of mutations that alter the drug targets. In Gram-negative bacteria, DNA gyrase is more susceptible to inhibition by quinolones than is topoisomerase IV, whereas in Gram-positive bacteria, topoisomerase IV is usually the prime target and DNA gyrase is intrinsically less susceptible. Consequently, resistant mutations are found in* gyrA* in Gram-negative bacteria and in* parC* in Gram-positive bacteria [26].

**Figure 5:**
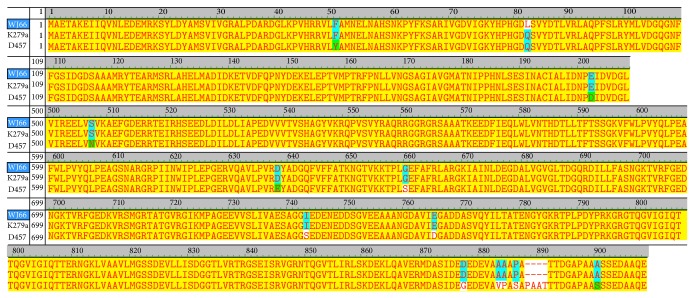
Alignment of the* gyrA* gene among* S. maltophilia* WJ66, K279a, and D457. Alignment of* gyrA* sequences of* S. maltophilia* WJ66, K279a, and D457 using vector NTI suite 9 software.

The MIC of levofloxacin for K279a is 1 *μ*g/mL, the MIC of norfloxacin for D457 is 6 *μ*g/mL [27], and the MIC of levofloxacin for WJ66 is more than 32 *μ*g/mL, corresponding to susceptibility results: sensitive, moderately sensitive, and resistant, respectively. Sequence alignments show that the resistant WJ66 strain contained a point mutation (Q83L) in its GyrA (Figure 5). While no mutations in GyrB were identified, WJ66 contained mutations in ParC (S86I and E421D) and ParE (M227L and F519C).

### 2.8. Efflux Pumps in WJ66 Genome

Efflux as a mechanism for antibiotic resistance was first described in 1980 [28]. In Gram-negative nosocomial pathogens, MDR is usually mediated by the overproduction of resistance-nodulation-division (RND) type efflux pumps. These pumps tend to have broad substrate profiles, including organic solvents, disinfectants, and antimicrobial drugs from a number of different classes. In the case of Gram-negative bacteria, efflux pumps consist of cytoplasmic, periplasmic, and outer membrane proteins (OMP) that associate to form multicomponent efflux systems [29]. A periplasm spanning membrane-fusion protein (MFP) is usually specific to each RND efflux protein, and it is common to find the pair encoded as part of an operon. A third component, the outer membrane protein, can be encoded in the same operon, but there tends to be fewer OMPs than RND/MFP pairs in a cell, meaning that the OMPs are often promiscuous [10]. The K279a sequence encodes nine RND-type efflux pump genes that fall into the drug resistance type based on sequence homology. WJ66 also possessed these efflux pumps (Table 3).

### 2.9. The Pathogen-Host Interactions Database (PHI-base)

The PHI-base (http://www.phi-base.org/) contains expertly curated molecular and biological information on genes proven to affect the outcome of pathogen-host interactions. Information is also given on the target sites of some anti-infective molecules.

Little is known about pathogenesis of* S. maltophilia* and the genomic diversity exhibited by clinical isolates complicates the study of pathogenicity and virulence factors. A recent study about relative virulence in adult-zebrafish model of* S. maltophilia* infection revealed that abundance of the quorum-sensing factor Ax21 in four strains of* S. maltophilia* correlates with mortality [30]. ORFs obtained from the GeneMarks annotation were subjected to BLAST analysis against the PHI-base, resulting in the identification of 252 homologous genes. Two genes,* ax21* and* avrXa21*, were found to be homologous to* Xanthomonas oryzae* genes that are related to plant virulence. The* ax21* and* avrXa21* genes also have homologs in* Metarhizium anisopliae* and* Saccharomyces cerevisiae*. Seven genes were annotated as lethal to host (plant); the first two have homologs in* Gibberella zeae*, and the other five have homologs in* Aspergillus fumigatus*. Seven genes were annotated to be resistant to chemical reagents including disinfectants and chemotherapy. Seven genes were annotated with mixed outcomes. A majority of genes, approximately 26, were annotated as partial loss of Ax21 signalling in this opportunistic pathogen strain, these genes include* raxB*,* raxC*,* raxH*,* raxR*,* raxH*_*2*_ */phoQ*, and* raxH*_*2*_ */phoP*, which has a homolog in* Xanthomon oryzae*. Thirty-three genes were annotated as genes that do not affect pathogenicity, while 42 genes and 125 genes were annotated as genes that lead to pathogenic loss and virulence attenuation, respectively. While a majority of pathogenic genes were avirulent, foreign outbreaks are examples of pathogenicity and virulence still worthy of our attention.

The rice XA21 receptor binds the AxYS22 peptide corresponding to the N-terminal region of Ax21, a type I-secreted protein that is highly conserved in all* Xanthomonas* species as well as in* Xylella fastidiosa* and* S. maltophilia* [31].

Ax21 is a 194-amino-acid protein and an activator of innate immunity mediated by the XA21 pattern recognition receptor [32].* S. maltophilia* displayed Ax21 (Smlt0378 in K279a) functions in the signal between cells. An Ax21 knockout leads to decreased virulence but a lack of raxST sulphated species modifications that do not affect the signal. These findings suggest that sulfation is not required for the regulatory action of Ax21 within* S. maltophilia* K279a [33]. Ax21 associated genes in* S. maltophilia* WJ66 are shown in Table 4. Homology analysis showed that WJ66 lacks* raxST* and* raxA*, which is consistent with K279a. WJ66_03588 is homologous to the* raxB* gene, a 169-amino-acid protein that functions in Colicin V secretion and had 97.78% homology to an ABC transporter ATP-binding protein. EPM1 predicted that Smlt2001 from K279a is a putative 590-amino-acid ABC transporter ATP-binding protein that has high homology to PHI_1141 (719 amino acids). Ax21 secretion depends on the RaxB homologous protein Smlt2001. Because of the size difference, confirming WJ66_03588 functions as Smlt2001 will need further verification. Additionally, raxP is missing in WJ66, and raxQ has only 37.04% homology.

## 3. Conclusions

In summary, the genomic sequence of the resistant bacteraemia-associated* S. maltophilia* isolate WJ66 is highly homologous with K279a. Tiny differences in nucleic acid levels lead to different* in vitro* antibiotic susceptibility phenotypes. Comparative genomic analyses revealed a considerable diversity among* S. maltophilia* strains, providing new insights into the differences and similarities that may explain the diverse nature of these strains. Drug-resistant gene mutations, drug-resistant enzymes, and horizontal drug resistance gene transfer in bacteria contribute to drug resistance. We obtained useful information about the unexplored aspects of* S. maltophilia*. The work will improve our understanding of the correlation between pathogenic traits and specific genes or gene clusters of* S. maltophilia*.

## 4. Material and Methods

### 4.1. Bacterial Strains,* In Vitro* Antimicrobial Sensitivity Assays, and Genomic DNA Extraction

*S. maltophilia* WJ66 was originally isolated in a blood sample from an elder male patient undergoing chemotherapy. The patient has diabetic nephropathy, chronic renal insufficiency, uraemia, renal anaemia, lung infection, hypertension, cerebral infarction (old), urinary tract infection, and hyperuricemia and did not respond to therapy with piperacillin-tazobactam, ceftazidime, or imipenem. The bacterium was performed the antimicrobial susceptibility assay and the MLST sequencing as previously described [34].

Susceptibility assays for the different antibiotics were performed in Mueller Hinton (MH) broth, using the twofold dilution method in 96-well microporous plates. The results were recorded after 20 h incubation at 37°C [35]. MICs were determined in MH medium by Etest (AB BIOMERIEUX, Arizona City, Sweden), according to the manufacturer's instructions. To ensure that the observed changes were consistent, all MICs were determined in three independent assays, using different bacterial cultures on different days. In most cases, there were no interassay differences in the MIC values. In a few cases, there were one dilution differences. For the latter, the assay was repeated one more time to further assure assay reliability. Control strains were* Escherichia coli* (ATCC25922) and* Pseudomonas aeruginosa* (ATCC27853). Genomic DNA was extracted using Invitrogen PureLink Genomic DNA Mini Kit according to the manufacturer's instructions.

### 4.2. Sequence Assembly, Prediction, and Annotation

After the DNA was prepared, a whole-genome shotgun (WGS) sequencing strategy and Illumina HiSeq 2000 sequencing technology with a paired-end protocol was used to determine the genome sequence of* S. maltophilia* WJ66. The low-quality reads were filtered, and the remaining reads were assembled into contigs with Velvet by the* de novo* assembly method. ORFs were predicted by the GeneMark analysis. The rRNA was predicted by using RNAmmer and the tRNA was identified with tRNA scan-SE. Then, the genomic sequence was annotated automatically using blast software for searching the SwissPort, UniPort, and the NR database; the cutoff of* E*-value was 1*e* − 5.

### 4.3. Nucleotide Sequence Accession Number

This entire genome shotgun project has been deposited in the DDBJ/EMBL/GenBank under the accession AZRF00000000. The version described in this paper is the first version, accession AZRF00000000.

### 4.4. Phylogenetic Analysis

After a maximum likelihood phylogenetic analysis, we construct a neighbour-joining phylogenetic tree with one single copy gene family. Sequences from* Xanthomonas campestris* pv.* campestris* strain 8004 were used as an outgroup.

### 4.5. Genome Alignments of* S. maltophilia* WJ66 and K279a

Annotated genome sequences and statistics of strain K279a from GenBank can be accessed through genome. Line figures depict the whole genome alignment results using MUMmer [19, 36]. The query genomic sequence was the strain WJ66 and the subject genome was K279a. The red and blue lines represent direct and reverse alignments, respectively.

## Supplementary Material

Table S1 Class 1 integron within S. maltophilia WJ66 genome.

## Figures and Tables

**Table 1:** Etest results.

Isolate	MIC (*μ*g/mL) of					
	IPM	TC	LE	TS	DC	PO
K279a	>32	32	1	0.125	16	1
WJ66	>32	>256	>32	>32	48	4
ATCC27853	1	32	0.5	32	64	2
ATCC25922	0.19	1.5	0.016	0.047	1.5	0.5

IPM: imipenem; TC: tetracycline; LE: levofloxacin; TS: sulfamethoxazole-trimethoprim; DC: doxycycline; PO: polymyxin.

**Table 2:** *S. maltophilia* WJ66 genome assembly results.

Type	WJ66 genome
Scaffold number	75
Scaffold length (bp)	4,657,282
Scaffold average length (bp)	62,097
Scaffold N50 (bp)	143,182
Contigs number	258
Contigs length (bp)	4,642,973
Contigs average length (bp)	17,996
Contigs N50 (bp)	33,808
Contigs G + C content (%)	66.48
ORFs number	4,392
ORFs length (bp)	4,118,607
ORFs average length (bp)	937
ORFs G + C content (%)	66.88
tRNA number	61
rRNA number	3

**Table 3:** Characteristics of Sme efflux transporters in *S. maltophilia *WJ66.

Name	Systematic ID	Known or putative regulation mechanism	Systematic ID
SmeABC	WJ66_02013, WJ66_02012, WJ66_02011	Two component regulator smeSR	WJ66_02014, WJ66_02015

SmeDEF	WJ66_02297, WJ66_02296, WJ66_02294	TetR type transcriptional regulator, smeT	WJ66_02298

SmeVWX	WJ66_01718, WJ66_01720, WJ66_01721	HTH-type transcriptional regulator	WJ66_01724

SmeYZ	WJ66_04223, WJ66_04224	Two component regulator	WJ66_02878, WJ66_02879

SmeGH	WJ66_01237, WJ66_01238	TetR type transcriptional regulator	WJ66_01236

SmeMN	WJ66_02576, WJ66_02575		

SmeOP	WJ66_00263, WJ66_00264	TetR type transcriptional regulator	WJ66_00262

SmeIJK	WJ66_03576, WJ66_03575, WJ66_03574		

**Table 4:** Ax21 associated genes in *S. maltophilia *WJ66.

Systematic ID	S. name	% identity 1	Gene name	Phenotype	% identy 2	Product-NR
WJ66_02052	PHI_1146	59.32	Ax21	Effector (plant avirulence determinant)	100	Putative uncharacterized protein (precursor) (strain K279a)

WJ66_01677	PHI_1146	53.67	Ax21	Effector (plant avirulence determinant)	95.81	Putative uncharacterized protein (precursor) (strain K279a)

WJ66_03588	PHI_1141	54.55	raxB	Partially lost Ax21 (synonym: AvrXa21) activity	97.78	Colicin V secretion ABC transporter ATP-binding protein (strain EPM1)

WJ66_00260	PHI_1142	71.26	raxC	Partially lost Ax21 (synonym: AvrXa21) activity	100	Putative outer membrane protein (precursor) (strain K279a)

WJ66_03632	PHI_1136	80.79	raxR	Partially lost Ax21 (synonym: AvrXa21) activity	100	Putative two-component system response regulator (strain K279a)

WJ66_03633	PHI_1137	58.11	raxH	Partially lost Ax21 (synonym: AvrXa21) activity	99.53	Putative two-component system sensor histidine (strain K279a)

WJ66_03508	PHI_1144	98.21	raxR2/phoP	Partially lost Ax21 (synonym: AvrXa21) activity/affected bacterial pathogenicity	100	Putative two-component system response regulator (strain K279a)

WJ66_03509	PHI_1145	77.07	raxH2/phoQ	Partially lost Ax21 (synonym: AvrXa21) activity	100	Putative two-component regulator sensor histidine kinase transmembrane transcriptional regulatory protein (precursor) (strain K279a)

WJ66_04429	PHI_1139	37.04	raxQ	Partially lost Ax21 (synonym: AvrXa21) activity	98.86	Elongation factor Tu (strain K279a)

% identity 1 and % identity 2 values were results from the identity when subjected to BLAST analysis against PHI database and NR database, respectively.
