# Supplementary material for: Identification and Characterization of a Serious Multidrug Resistant Stenotrophomonas maltophilia Strain in China
Source: Biomed Res Int. 2015 Jan 14;2015:580240. doi: 10.1155/2015/580240 (PMC4310304; doi:10.1155/2015/580240)
Supplement: Supplementary file 1 — Table S1 Class 1 integron within S. maltophilia WJ66 genome. [file 580240.f1.pdf]

Supplementary data

Table S1 Class 1 integron within *S. maltophilia* WJ66 genome

Seq_ID	Start	End	Strand	Color	Gene_Name	Annotation
contig_00009	44125	46041	+	Blue	3’CS	
contig_00009	44125	44625	-	Green	orf5	hypothetical protein
contig_00009	44753	45592	-	Green	sul1	sulfonamide resistance protein
contig_00009	45586	45933	-	Green	qacED1	quarternary ammonium coumpounds resistance protein
contig_00009	46097	46888	-	Green	aadA2	aminoglycoside adenyltransferase
contig_00009	46898	47831	+	Blue	5’CS	
contig_00009	47034	47831	+	Orange	intI1	Integron integrase IntI1
contig_00009	47884	48588	-	Green	tnpA	transposase
